# Effects of ergosteroside combined risedronate on fracture healing and BMP-2, BMP-7 and VEGF expression in rats

**DOI:** 10.1590/ACB361107

**Published:** 2021-12-17

**Authors:** Xiaofeng Xu, Wenyu Hui, Nian Liu, Yong Zhang

**Affiliations:** 1 MD. Department of Oral & Cranio-maxillofacial Surgery - Shanghai Ninth People’s Hospital - Shanghai Jiao Tong University School of Medicine - Shanghai - China.; 2 MDS. Department of Oral & Cranio-maxillofacial Surgery - Shanghai Ninth People’s Hospital - Shanghai Jiao Tong University School of Medicine - Shanghai - China.; 3 PhD. Department of Oral & Cranio-maxillofacial Surgery - Shanghai Ninth People’s Hospital - Shanghai Jiao Tong University School of Medicine - Shanghai - China.; 4 DDS, MD. Department of Oral & Cranio-maxillofacial Surgery - Shanghai Ninth People’s Hospital - Shanghai Jiao Tong University School of Medicine - Shanghai - China.

**Keywords:** Risedronic Acid, Fracture Healing, Bone Morphogenetic Protein-2, Bone Morphogenetic Protein-7, Vascular Endothelial Growth Factor, Rats

## Abstract

**Purpose:**

To evaluate the effect of ergosterol combined with risedronate on fracture healing.

**Methods:**

Sixty male Sprague Dawley fracture model rats were assigned into group A (n=20), group B (n=20), and group C (n=20) at random. All rats were fed by gavage until their sacrifice as it follows: group A with ergosteroside and risedronate, group B with risedronate, and group C with saline solution. At weeks 2 and 4, 10 rats of each group were sacrificed. Healing effect and bone tissue changes in the fractures site were assessed by using hematoxylin and eosin stain histology. Enzyme-linked immunosorbent assay was used to detect the expression of serum bone morphogenetic protein-2 (BMP-2), bone morphogenetic protein-7 (BMP-7), and vascular endothelial growth factor (VEGF). Reverse transcriptase polymerase chain reaction was applied to detect the expression of osteoprotegerin (OPG) mRNA, osteocalcin (OCN) mRNA and core-binding factor subunit-?1 (CBF-?1) mRNA.

**Results:**

In terms of serum BMP-2, BMP-7, and VEGF expression at weeks 2 and 4 after gavage, group A < group B < group C (P<0.05). At week 4 after gavage, serum VEGF expression in the three groups harbored positive relationship with serum BMP-2 and BMP-7 expression (P<0.05). Regarding serum OPG, OCN and CBF-?1 mRNA expression at weeks 2 and 4 after gavage, group A <group B <group C (P<0.05). Hematoxylin and eosin staining results showed that the recovery effect of trabecular bone and callus in the cases of group A was better than the other two groups after intragastric administration.

**Conclusion:**

Ergosteroside combined risedronate can patently ameliorate the healing effect of fracture in rats.

## Introduction

Fracture refers to the continuous partial or complete fracture of bone structure, which can occur in people of any age^1^. Elderly patients with fractures are principally due to falls outside, coupled with the older age group, the bones are relatively weak and fragile, while young patients are mostly induced by serious accidents such as car accidents and falls from heights^2^,^3^. The clinical symptoms of fracture patients are primarily local symptoms, such as pain, bleeding, restricted activity, deformity and systemic symptoms such as fever and shock at the fracture site, which can have varying degrees of impact on the quality of life and life safety of patients^4^.

Kui *et al*.^5^ have corroborated that the prognosis of fracture patients is compactly connected to the expression of multiple bone growth factors in the body. Bone morphogenetic protein-2 (BMP-2) and bone morphogenetic protein-7 (BMP-7) are the cardinal bone morphogenetic proteins in the body. Mediated and promoted bone formation alone can also facilitate bone formation by combining with other bone growth factors^6^. Vascular endothelial growth factor (VEGF) can influence the prognosis of fracture patients by modulating the level of inflammatory activity in the body^7^.

Ergosteroside, a kind of phenethanol glycoside monomer compound, harbors perspicuous effects of meliorating fracture healing and analgesia. In recent years, it has been extensively applied in the clinical treatment of patients with orthopedic diseases^8^. Risedronate can curb osteoclasts and bone resorption by combining with hydroxyapatite in bone, which is often utilized clinically in the treatment of fractures and osteoporosis patients^9^. Nevertheless, in recent years, studies on the combined application of the two in the treatment of fracture patients are lacking and scanty, meanwhile the mechanism of action has not yet been demystified.

Hereby, this study fabricated a fracture rat model and gave different treatment methods, aiming to delve into the healing effect of ergosteroside combined risedronate on rat fracture healing and the influence on BMP-2, BMP-7, and VEGF expression.

## Methods

The present study was approved by the Ninth People’s Hospital Animal Experimental Ethics Committee (Shanghai, China).

Sixty healthy male Sprague Dawley rats, 8-10 weeks old, body weight (187.52±5.16) g, from Beijing Baoyuan Xingye Technology Co., Ltd., were used.

### Medicines, instruments, and equipment

Ergosteroside (Chengdu Purechem-standard Co., Ltd., Chengdu, China); risedronate tablets (Jiangsu Chiatai Qingjiang Pharmaceutical Co., Ltd., Huaian, Chain, H20100136); 0.9% sodium chloride solution (Shandong Qidu Pharmaceutical Co., Ltd., Zibo, China, H20113297); high-frequency mobile C-arm X-ray machine (Nanjing Perlove Medical Equipment Co., Ltd.); hematoxylin and eosin (HE) staining reagents and solutions (Beijing Solarbio Science & Technology Co., Ltd.); mouse enzyme-linked immunosorbent assay (ELISA) detection reagents and kits (Shanghai Yanyu Chemical Reagent Co., Ltd.); osteoprotegerin (OPG) mRNA, osteocalcin (OCN) mRNA, core-binding factor subunit-α1 (CBF-α1) mRNA and U6 primer (Shanghai Sangon Biotech Co., Ltd.) were used (Table 1).

**Table 1:** Primer sequence of OPG mRNA, OCN mRNA, CBF-α1 mRNA and U6.

Proteins	Upstream primer	Downstream primer
OPG mRNA	5’-ACACTCCAGCTCCCCTTCTCCTGGCTCTCCT-3’	5’-TGGTGTCGTCCAGTCG-3’
OCN mRNA	5'-GGCGGTGCTCGCTTTGTA-3'	5'-TCCCGAATGTCTGACGTATTGA-3 '
CBF-α1 mRNA	5'-CGAGAACACTAACTTC-CCCGC-'	5'-GTGGTTCATCTGGTGGTCGC-TA-3'
U6	5'-TTCCTACCCCCAATGTATCCG-3'	5'-CATGAGGTCCACCACCCTGTT-3'

OPG: osteoprotegerin; OCN: osteocalcin; CBF-α1: core-binding factor subunit-α1.

### Model construction

After weighing, 60 rats received general anesthesia. After the anesthesia was fully effective, their left lower limbs were prepared and disinfected. The skin near the left mandible was cut and separated to completely expose the bone tissue. Emery tablets were applied to the left mandible. We made a bone defect area with a width of 1 mm and a length of about 3 mm on the lower edge of the bone and took care not to damage the bone defect areas in other parts near the bone defect area. The wound area was disinfected utilizing penicillin, conventional sutures were fulfilled, and the incision was closed^10^. Three days after surgery, the model was successful if the X-ray examination showed obvious bone defect.

### Grouping and administration

Sixty rats were randomly allocated into group A (n=20), group B (n=20), and group C (n=20). Drug intervention was implemented on day 5 after surgery. Group A was given 0.8% ergosteroside and risedronate by gavage with a dose of 5 mL/kg for four weeks. Group B was given 0.6% risedronate solution by gavage with a dose of 3 mL/kg. Group C was given 0.9% sodium chloride solution by gavage.

### Specimen collection and index testing

#### Radiological examination

Ten rats in each group were sacrificed two and four weeks after gavage, and the mandibles were taken out immediately after death for X-ray photography, in order to observe the fracture healing of the three groups of rats.

#### ELISA detection

Before the rats were sacrificed, blood was taken from the heart and kept for 30 min. After the whole blood had spontaneously coagulated and the serum was precipitated, the supernatant was obtained by centrifugation at about 1,000-2,000 g lasting 10 min at 4°C. Serum BMP-2, BMP-7 and VEGF expressions were assayed via ELISA. We set blank wells, standard wells, and sample wells. We added 50 μL of standards with different concentrations to standard wells, added 10 μL of samples to the sample wells, then added 40 μL of sample diluent. In addition to the blank wells, we added 100 μL of horseradish peroxidase-labeled detection antibody to each well of the standard and sample wells, sealed the reaction wells with a sealing film, incubated it in a 37°C water bath lasting 65 min, discarded the liquid, patted dry on absorbent paper, filled each well with washing solution, allowed to stand for 2 min, shake off washing solution, patted dry on absorbent paper, and repeated six times. Subsequently, we added 50 μL each of substrates A and B to each well, incubated it at 37°C lasting 10 min in the dark, added 50 μL of stop solution to each well, measured the optical density (OD) value of each well at 450 nm within 15 min, and finally calculated the concentration^11^.

#### Reverse transcriptase polymerase chain reaction detection

In order to extract RNA from bone tissue, the bone tissue was crushed with bone forceps and put into a mortar. After drying for 2 hours at 180°C, liquid nitrogen was added and repeatedly ground into fine powder, and then cracking fluid was added and blown into tissue homogenate. OPG, OCN and CBF-α1 mRNA expression in rat bone tissue were assayed via polymerase chain reaction (PCR) technology. We made use of Trizol for extracting total RNA from tissue, as well as reverse transcriptase (RT) and oligonucleotides for synthesizing cRNA according to the operating instructions.

- Transcription reaction system (20 μL): buffer 4 μL, RT 2 μL, total RNA 2 μL, and RNase water 12 μLl
- Reaction conditions: at 42°C in a water bath lasting 1 h, at 95°C in a water bath lasting 5 min. PCR machine was used for amplification reaction, with RNU6B as an internal reference control. We detected OPG, OCN and CBF-α1 mRNA expressions using their specific primers in a fluorescent quantitative PCR instrument according to the operating instructions;
- PCR reaction system (20 μL): 0.4 μL of upstream primer, 0.4 μL of downstream primer, 0.5 μL of miR, and the rest was filled with ddH2O;
- RT-PCR conditions: 94°C for 10 s, 94°C for 5 s, 52°C for 30 s annealing, 72°C for 15 s, and then 40 cycles. Each experiment set three duplicate holes. We repeated the experiment three times. The experimental results were analyzed via relative quantitative method^12^.

#### HE staining

- Put the deparaffinized rat bone tissue section into water for dyeing for several minutes;
- Put the section into acid water and ammonia water for color separation, each counts for 5-8 s;
- After rinsing with flowing water lasting 1 h, put in distilled water for a while;
- Dehydrate in 70 and 90% alcohol lasting 10 min each;
- Stain with alcohol eosin staining solution lasting 2-3 min;
- Dehydrate the stained sections with pure alcohol. Xylene makes the section transparent;
- Drip the transparent section with Canadian gum and cover it with a cover glass for sealing^13^.

### Statistical analysis

Making use of Statistical Package for the Social Sciences (SPSS) 25.0 software, we conducted statistical analysis, in which the measurement data were exhibited as mean ± standard deviation (`x±s), and the comparison between the three groups was analyzed via analysis of variance (ANOVA). Logistic regression was used for analyzing the relationship between indicators. *P<0.05. We used the GraphPad Prism 7.0 software for mapping.

## Results

### Comparison of serum BMP-2, BMP-7, and VEGF expressions after gavage

**Figure 1:**
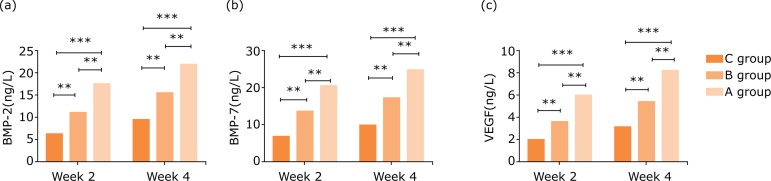
Comparison of serum BMP-2, BMP-7, VEGF, and IL-6 expressions after gavage. **(a)** BMP-2; **(b)** BMP-7; **(c)** VEGF.

BMP-2: bone morphogenetic protein-2; BMP-7: bone morphogenetic protein-7; VEGF: vascular endothelial growth factor.

At weeks 2 and 4 after gavage, serum BMP-2, BMP-7, and VEGF expression in the three groups of rats were group A <group B <group C, with statistical significance (P<0.05) (Table 2, Fig. 1).

**Table 2:** Comparison of serum BMP-2, BMP-7, VEGF and IL-6 expressions after gavage (`x±s, ng/L)*.

Serum	Week	Group A(n=10)	Group B(n=10)	Group C(n=10)	F	*P*
BMP-2	Week 2	17.43±3.32	11.25±2.26	6.37±1.69	6.738	0.001
	Week 4	22.06±5.93^a^	15.53±3.74^a^	9.42±2.17^a^	7.254	0.001
BMP-7	Week 2	20.31±4.56	13.72±2.59	7.28±1.84	6.492	0.001
	Week 4	24.57±6.29^a^	17.15±3.92^a^	10.13±2.04^a^	6.387	0.001
VEGF	Week 2	6.07±0.53	3.76±0.48	2.04±0.32	6.415	0.001
	Week 4	8.23±0.75^a^	5.53±0.62^a^	3.19±0.41^a^	7.173	0.001

* *vs.* week 2;

a P<0.05;

BMP-2: bone morphogenetic protein-2; BMP-7: bone morphogenetic protein-7; VEGF: vascular endothelial growth factor.

### Comparison of the correlation of serum VEGF with BMP-7 and BMP-2

**Figure 2:**
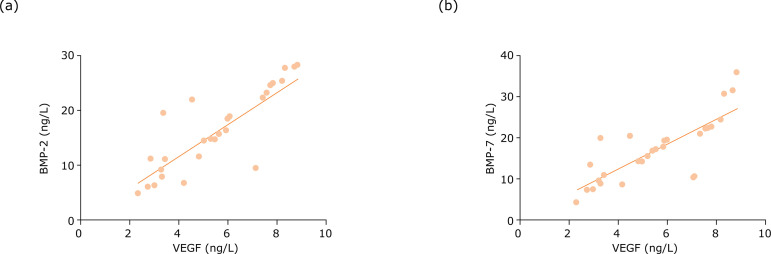
Comparison of the correlation of serum VEGF with BMP-7 and BMP-2 at week 4 after gavage. **(a)** Serum VEGF and serum BMP-2 are positively correlated, r=0.638, P=0.001; **(b)** Serum VEGF and serum BMP-7 are positively correlated, r=0.614, P=0.001.

BMP-2: bone morphogenetic protein-2; BMP-7: bone morphogenetic protein-7; VEGF: vascular endothelial growth factor.

At week 4 after gavage, serum VEGF expression in the three groups possessed positive relationship with serum BMP-2 and BMP-7 expressions (P<0.05) (Fig. 2).

### Comparison of OPG, OCN and CBF-α1 mRNA expressions after gavage

**Figure 3:**
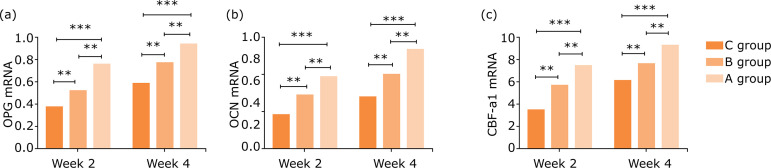
Comparison of OPG, OCN and CBF-α1 mRNA expressions after gavage. **(a)** OPG mRNA, **(b)** OCN mRNA, **(c)** CBF-α1 mRNA.

OPG: osteoprotegerin; OCN: osteocalcin; CBF-α1: core-binding factor subunit-α1.

In terms of OPG, OCN and CBF-α1 mRNA expressions in the three groups of rats at weeks 2 and 4 after gavage, group A <group B <group C, with statistical significance (P<0.05) (Table 3, Fig. 3).

**Table 3:** Comparison of OPG, OCN and CBF-α1 mRNA expressions after modeling (`x±s)*.

Proteins	Week	A(n=10)	B(n=10)	C(n=10)	F	*P*
OPG mRNA	Week 2	0.76±0.17	0.52±0.15	0.37±0.11	6.274	0.001
	Week 4	0.94±0.25^a^	0.77±0.19^a^	0.58±0.14^a^	5.736	0.003
OCN mRNA	Week 2	0.65±0.21	0.49±0.17	0.32±0.14	5.954	0.002
	Week 4	0.89±0.27^a^	0.67±0.23^a^	0.48±0.18^a^	6.382	0.001
CBF-α1 mRNA	Week 2	0.74±0.19	0.58±0.16	0.35±0.14	5.412	0.005
	Week 4	0.93±0.26^a^	0.77±0.21^a^	0.61±0.17^a^	5.695	0.003

OPG: osteoprotegerin; OCN: osteocalcin; CBF-α1: core-binding factor subunit-α1;

* week 2;

a P<0.05.

### HE staining results of bone tissue in the three groups of rats after gavage

**Figure 4:**
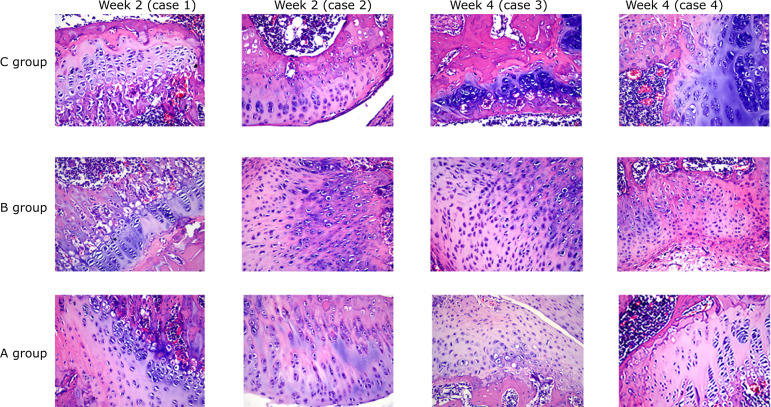
Hematoxylin and eosin staining results of bone tissue in fracture rats.

HE staining displayed that the number of trabeculae in the three groups were saliently less with disordered arrangement at week 2 after gavage. However, the number of new bone trabeculae in the rats in group A was more than that in the other two groups. At week 4 after gavage, the trabecular bone of the rats in group A became thicker, and the callus remodeled obviously, which was similar to the nearby trabecular bone, but the arrangement was still disordered. The trabecular bone remodeling of the rats in groups B and C was not obvious, the thickness was different, the distribution was sparse, and the fracture was visible. The restoration effect of trabecular bone and callus of the rats in group A was outstandingly better than that of the other two groups (Fig. 4).

## Discussion

In recent years, with the continuous acceleration of the aging process and the frequent occurrence of traffic accidents and accidents in general, the number of clinical fracture patients has increased blatantly, and there is a gradual younger trend^14^,^15^. The clinical treatment methods of fracture patients dominatingly include surgery, drug therapy, and physical therapy. Among them, drug therapy is dominantly suitable for patients with minor fractures or combined with other treatment methods, which can availably promote fracture healing of patients^16^,^17^. Nonetheless, the effects of varying drugs in facilitating fracture healing are quite distinct, and the mechanism of action of related drugs has not been fully expounded in clinical practice^18^. Both risedronate and ergosteroside are prevailingly utilized clinical drugs for fracture patients. This study probed the application value of the combination of the two in rat fractures and its mechanism of action.

Risedronate is currently a prevalent clinical drug for postmenopausal osteoporosis patients, and it can also be employed in the therapy of fracture patients^19^. In mechanism, risedronate is combined with hydroxyapatite in normal bone tissue to curb bone absorption and osteoclast formation^20^. Pertinent studies have authenticated that risedronate can not only sensibly reduce the bone turnover rate and bone resorption rate of patients with osteoporosis or fractures, but also noticeably reduce the absorption rate of the bone remodeling site, ultimately accelerating bone and bone structure to return to normal.

Ergosteroside is the predominant component of Chinese herbal medicines such as *Cistanche*. Traditional Chinese medicine states that *Cistanche* owns the effects of building up muscles and bones, tonifying kidney and strengthening yang. In recent years, *Cistanche* has been broadly employed in the clinical treatment of patients with fractures and osteoporosis. As the prime component of *Cistanche*, ergosteroside has the functions of enhancing immunity and memory, lowering blood fat, relieving pain, and laxative on the one hand, and furthering fracture healing on the other hand^21^. In recent years, clinical related animal experiments have evinced that ergosteroside can forward fracture healing by facilitating the proliferation of T lymphocytes and osteoblasts^22^.

The influence of fracture healing is tightly concerned with diverse cytokines and signal transduction pathways in the body^23^. BMP-2 and BMP-7 are the staple bone morphogenetic proteins in the body. Both can stimulate DNA synthesis and cell replication, promote the differentiation of bone marrow stromal cells (BMSCs) into osteoblasts, expand the proliferation of osteoblasts, induce the formation of internal cartilage and bone, and participate in and control the embryonic development and repair and reconstruction process of bones^24^. VEGF can participate in the reconstruction of vascular tissue, growth and development and the formation of fibrous callus after a fracture by binding to specific receptors of endothelial blood vessels, and ultimately contribute to the proliferation of zygomatic cells and bone cells. Also, VEGF can take part in the process of fracture healing via adjusting BMP-2 and BMP-7 expressions in the body^25^,^26^. Serum BMP-2, BMP-7 and VEGF are all momentous factors that govern the impact of fracture healing. Martijn *et al*. have evidenced that serum BMP-2, BMP-7, and VEGF expressions in rats with poor fracture healing were prominently lower than that of rats with better healing^27^. In this research, the serum BMP-2, BMP-7, and VEGF expressions of rats in group A were eminently higher than those in the other two groups after gavage. X-ray examination and HE staining uncovered that the fracture healing and trabecular bone and callus recovery of rats in group A were preeminently better than the other two groups, implying that risedronate combined ergosteroside might increase serum BMP-2, BMP-7 and VEGF expressions in rats to promote fracture healing. Both risedronate and ergosteroside can compellingly reduce the bone resorption rate and contribute to the proliferation and differentiation of osteoblasts. The combined use of the two can not only exert synergistic functions between different drugs, but also boost their respective efficacy, finally contributing to the healing of rat fractures. In our findings, serum VEGF expression in group A harbored positive association with serum BMP-2 and BMP-7 expressions respectively at week 4 after gavage, illuminating that VEGF might participate in the fracture healing process of rats by controlling BMP-2 and BMP-7 expressions.

OPG, one of the leading members of the tumor necrosis factor (TNF) family, can restrain the proliferation and differentiation of osteoclasts by destroying the activity of osteoclasts, reduce the rate of bone resorption, participate in the modulation of bone metabolism, and foster the recovery of fractures in the body^28^. OCN is principally synthesized by osteoblasts and functions monumentally in modulating bone calcium metabolism. Its pathological decrease can be observed in many bone diseases such as osteoporosis, bone trauma, and bone tumors^29^. CBF-α1 is currently the latest clinically discovered osteoblast growth factor, and as a downstream factor of the BMP pathway, can directly act on downstream osteogenic genes^30^. Bae *et al*.^31^ have substantiated that compared with factors such as BMP-2 and BMP-7, the osteogenic effect of CBF-α1 is more telling and faster. In this research, OPG, OCN, and CBF-α1 mRNA expressions in group A after gavage were prominently higher than those of the other two groups, hinting that risedronate combined ergosteroside could dramatically promote OPG, OCN and CBF-α1 mRNA expressions in fracture rats, and eventually meliorate the fracture healing effect.

## Conclusions

Ergosteroside combined risedronate can substantially ameliorate the healing effect of rat fractures and expedite BMP-2, BMP-7, and VEGF expressions to return to normal. There are still some shortcomings in this work. For instance, we only used rat experiments to dig into the application value of ergosteroside combined risedronate in fractured organisms. In the future, we can treat clinical patients with related drugs for better observation of the application value of the two in the human body, to provide reference and rationale for the medication of clinical fracture patients.

## References

[1] Meinberg E, Agel J, Roberts C, Karam M, Kellam JF (2018). Fracture and dislocation classification
compendium-2018. J Orthop Trauma.

[2] Lin PP, Schupak KD, Boland PJ, Brennan MF, Healey JH (1998). Pathologic femoral fracture after periosteal excision and
radiation for the treatment of soft tissue sarcoma. Cancer.

[3] Ghoshal A, Enninghorst N, Sisak K, Balogh ZJ. (2018). An interobserver reliability comparison between the Orthopaedic
Trauma Association’s open fracture classification and the Gustilo and
Anderson classification. Bone Joint J..

[4] Tutton E, Achten J, Lamb SE, Willett K, Costa ML (2018). A qualitative study of patient experience of an open fracture of
the lower limb during acute care. Bone Joint J.

[5] Huang K, Wu G, Zou J, Peng S. (2018). Combination therapy with BMP-2 and psoralen enhances fracture
healing in ovariectomized mice. Exp Ther Med.

[6] Yan X, Zhou Z, Guo L, Zeng Z, Guo Z, Shao Q, Xu W. (2018). BMP7-overexpressing bone marrow-derived mesenchymal stem cells
(BMSCs) are more effective than wild-type BMSCs in healing
fractures. Exp Ther Med..

[7] Li S, Yuan H, Pan J, Fan W, Zhu L, Yan Z, Guo C. (2017). The treatment of femoral neck fracture using VEGF-loaded
nanographene coated internal fixation screws. PLoS One.

[8] Li X, Xie Y, Li K, Wu A, Xie H, Guo Q, Xue P, Maleshibek Y, Zhao W, Guo J, Chen D. (2018). Antioxidation and cytoprotection of acteoside and its
derivatives: comparison and mechanistic chemistry. Molecules.

[9] Mukherjee D, Srinivasan B, Anbu J, Azamthulla M, Banala VT, Ramachandra SG. (2018). Improvement of bone microarchitecture in methylprednisolone
induced rat model of osteoporosis by using thiolated chitosan-based
risedronate mucoadhesive film. Drug Dev Ind Pharm.

[10] Zandi M, Dehghan A, Amini P, Doulati S, Rezaeian L. (2019). Evaluation of the effect of teriparatide therapy on mandibular
fracture healing in rats with medication-related osteonecrosis of the
jaw. Clin Oral Investig.

[11] Guo TZ, Wei T, Huang TT, Kingery WS, Clark JD (2018). Oxidative stress contributes to fracture/cast-induced
inflammation and pain in a rat model of complex regional pain
syndrome. J Pain.

[12] Sun J, Jing S, Jiang R, Wang C, Zhang C, Chen J, Li H. (2018). Metabolomics study of the therapeutic mechanism of Schisandra
chinensis lignans on aging rats induced by d-galactose. Clin Interv Aging.

[13] Li CW, Liang B, Shi XL, Wang H. (2015). Opg/Rankl mRNA dynamic expression in the bone tissue of
ovariectomized rats with osteoporosis. Genet Mol Res.

[14] Bhavsar MB, Han Z, DeCoster T, Leppik L, Costa Oliveira, Barker JH. (2020). Electrical stimulation-based bone fracture treatment, if it works
so well why do not more surgeons use it?. Eur J Trauma Emerg Surg.

[15] Misir A, Ozturk K, Kizkapan TB, Yildiz KI, Gur V, Sevencan A. (2018). Fracture lines and comminution zones in OTA/AO type 23C3 distal
radius fractures: the distal radius map. J Orthop Surg (Hong Kong).

[16] Decheng W, Hao S, Zhongwei W, Jiaming L, Bin Y, Yong H (2019). Three-step reduction therapy of integrated chinese and western
medicine for thoracolumbar burst fracture. J Invest Surg.

[17] Hamilton K, Josiah DT, Tierney M, Brooks N. (2019). Surgical practice in traumatic spinal fracture treatment with
regard to the subaxial cervical injury classification and severity and the
thoracolumbar injury classification and severity systems: a review of 58
patients at the University of Wisconsin. World Neurosurg.

[18] Rehm A, Thahir A. (2020). Regionalization of isolated pediatric femur fracture treatment:
recent trends observed using the kids’ inpatient database. J Pediatr Orthop.

[19] Reshamwala SMS, Mamidipally C, Pissurlenkar RRS, Coutinho EC, Noronha SB. (2016). Evaluation of risedronate as an antibiofilm agent. J Med Microbiol.

[20] Santhosh S, Mukherjee D, Anbu J, Murahari M, Teja BV (2019). Improved treatment efficacy of risedronate functionalized
chitosan nanoparticles in osteoporosis: formulation development, in vivo,
and molecular modelling studies. J Microencapsul.

[21] Peng XM, Gao L, Huo SX, Liu XM, Yan M. (2015). The mechanism of memory enhancement of acteoside (verbascoside)
in the senescent mouse model induced by a combination of D-gal and
AlCl3. Phytother Res.

[22] Pesce M, Franceschelli S, Ferrone A, De Lutiis, Patruno A, Grilli A, Felaco M, Speranza L (2015). Verbascoside down-regulates some pro-inflammatory signal
transduction pathways by increasing the activity of tyrosine phosphatase
SHP-1 in the U937 cell line. J Cell Mol Med.

[23] Fischer C, Reiner C, Schmidmaier G, Doll J, Child C, Grützner PA, Biglari B, Boxriker S, Moghaddam A (2018). Safety study: is there a pathologic IGF-1, PDGF and TGF-?
cytokine expression caused by adjunct BMP-7 in tibial and femoral non-union
therapy?. Ther Clin Risk Manag.

[24] Kawai M, Kataoka Y, Sonobe J, Yamamoto H, Maruyama H, Yamamoto T, Bessho K, Ohura K. (2018). Analysis of mineral apposition rates during alveolar bone
regeneration over three weeks following transfer of BMP-2/7 gene via in vivo
electroporation. Eur J Histochem.

[25] Sharma S, Sapkota D, Xue Y, Rajthala S, Yassin MA, Finne-Wistrand A, Mustafa K. (2018). Delivery of VEGFA in bone marrow stromal cells seeded in
copolymer scaffold enhances angiogenesis, but is inadequate for osteogenesis
as compared with the dual delivery of VEGFA and BMP2 in a subcutaneous mouse
model. Stem Cell Res Ther.

[26] Hofman M, Koopmans G, Kobbe P, Poeze M, Andruszkow H, Brink PR, Pape HC (2015). Improved fracture healing in patients with concomitant traumatic
brain injury: proven or not?. Mediators Inflamm.

[27] Huang B, Yao Q, Huang Y, Zhang L, Yao Y, Gong P, Tang H. (2018). Combination use of BMP2 and VEGF165 promotes osseointegration and
stability of titanium implants in irradiated bone. Biomed Res Int..

[28] Krzanowski M, Krzanowska K, Dumnicka P, Gajda M, Woziwodzka K, Fedak D, Grodzicki T, Litwin JA, Su?owicz W (2018). Elevated Circulating osteoprotegerin levels in the plasma of
hemodialyzed patients with severe artery calcification. Ther Apher Dial.

[29] Lin X, Parker L, McLennan E, Zhang X, Hayes A, McConell G, Brennan-Speranza TC, Levinger I. (2018). Uncarboxylated osteocalcin enhances glucose uptake ex vivo in
insulin-stimulated mouse oxidative but not glycolytic muscle. Calcif Tissue Int.

[30] Wattanavanitchakorn S, Rojvirat P, Chavalit T, MacDonald MJ, Jitrapakdee S. (2018). CCAAT-enhancer binding protein-? (C/EBP?) and hepatocyte nuclear
factor 4? (HNF4?) regulate expression of the human
fructose-1,6-bisphosphatase 1 (FBP1) gene in human hepatocellular carcinoma
HepG2 cells. PLoS One.

[31] Bae SJ, Kim HJ, Won HY, Min YK, Hwang ES (2017). Acceleration of osteoblast differentiation by a novel osteogenic
compound, DMP-PYT, through activation of both the BMP and Wnt
pathways. . Sci Rep.

